# Circulating tumor DNA integrating tissue clonality detects minimal residual disease in resectable non-small-cell lung cancer

**DOI:** 10.1186/s13045-022-01355-8

**Published:** 2022-10-01

**Authors:** Siwei Wang, Ming Li, Jingyuan Zhang, Peng Xing, Min Wu, Fancheng Meng, Feng Jiang, Jie Wang, Hua Bao, Jianfeng Huang, Binhui Ren, Mingfeng Yu, Ninglei Qiu, Houhuai Li, Fangliang Yuan, Zhi Zhang, Hui Jia, Xinxin Lu, Shuai Zhang, Xiaojun Wang, Youtao Xu, Wenjia Xia, Tongyan Liu, Weizhang Xu, Xinyu Xu, Mengting Sun, Xue Wu, Yang Shao, Qianghu Wang, Juncheng Dai, Mantang Qiu, Jinke Wang, Qin Zhang, Lin Xu, Hongbing Shen, Rong Yin

**Affiliations:** 1 Department of Thoracic Surgery, Jiangsu Key Laboratory of Molecular and Translational Cancer Research, Jiangsu Cancer Hospital &, Nanjing Medical University Affiliated Cancer Hospital & Jiangsu Institute of Cancer Research, Nanjing, 21009 People’s Republic of China; 2 Department of Pathology, Jiangsu Cancer Hospital & Nanjing Medical University Affiliated Cancer Hospital & Jiangsu Institute of Cancer Research, Nanjing, 21009 People’s Republic of China; 3 Department of Oncology, Jingxian Hospital, Hengshui, 053099 People’s Republic of China; 4 Nanjing Geneseeq Technology Inc., Nanjing, 210032 People’s Republic of China; 5 Department of Science and Technology, Jiangsu Cancer Hospital & Nanjing Medical University Affiliated Cancer Hospital & Jiangsu Institute of Cancer Research, Nanjing, 21009 People’s Republic of China; 6 Biobank of Lung Cancer, Jiangsu Biobank of Clinical Resources, Nanjing, 21009 People’s Republic of China; 7 Collaborative Innovation Center for Cancer Personalized Medicine, Nanjing Medical University, Nanjing, 211116 People’s Republic of China; 8 Department of Bioinformatics, Nanjing Medical University, Nanjing, 211166 People’s Republic of China; 9 Department of Biostatistics, Center for Global Health, School of Public Health, Nanjing Medical University, Nanjing, 211116 People’s Republic of China; 10 Department of Epidemiology and Biostatistics, International Joint Research Center On Environment and Human Health, Center for Global Health, School of Public Health, Nanjing Medical University, Nanjing, 211116 People’s Republic of China; 11 Department of Thoracic Surgery, Peking University People’s Hospital, Beijing, 100044 People’s Republic of China; 12 State Key Laboratory of Bioelectronics, Southeast University, Nanjing, 210018 People’s Republic of China

**Keywords:** Circulating tumor DNA, Minimal residual disease, Liquid biopsy, Non-small-cell lung cancer

## Abstract

**Background:**

Circulating tumor DNA (ctDNA) has been proven as a marker for detecting minimal residual diseases following systemic therapies in mid-to-late-stage non-small-cell lung cancers (NSCLCs) by multiple studies. However, fewer studies cast light on ctDNA-based MRD monitoring in early-to-mid-stage NSCLCs that received surgical resection as the standard of care.

**Methods:**

We prospectively recruited 128 patients with stage I–III NSCLCs who received curative surgical resections in our Lung Cancer Tempo-spatial Heterogeneity prospective cohort. Plasma samples were collected before the surgery, 7 days after the surgery, and every 3 months thereafter. Targeted sequencing was performed on a total of 628 plasma samples and 645 matched tumor samples using a panel covering 425 cancer-associated genes. Tissue clonal phylogeny of each patient was reconstructed and used to guide ctDNA detection.

**Results:**

The results demonstrated that ctDNA was more frequently detected in patients with higher stage diseases pre- and postsurgery. Positive ctDNA detection at as early as 7 days postsurgery identified high-risk patients with recurrence (HR = 3.90, *P* < 0.001). Our results also show that longitudinal ctDNA monitoring of at least two postsurgical time points indicated a significantly higher risk (HR = 7.59, *P* < 0.001), preceding radiographic relapse in 73.5% of patients by a median of 145 days. Further, clonal ctDNA mutations indicated a high-level specificity, and subclonal mutations informed the origin of tumor recurrence.

**Conclusions:**

Longitudinal ctDNA surveillance integrating clonality information may stratify high-risk patients with disease recurrence and infer the evolutionary origin of ctDNA mutations.

**Supplementary Information:**

The online version contains supplementary material available at 10.1186/s13045-022-01355-8.

## To the editor

Approximately 30–55% of non-small-cell lung cancer (NSCLC) patients developed recurrence despite curative resection [1]. Circulating tumor DNA (ctDNA) is shed by tumor cells and may serve as an effective prognostic marker following multiple therapeutic modalities [2–5]. However, it remained not fully understood to what extent serial ctDNA monitoring could help identify the risk of recurrence in resectable NSCLC.

**Fig. 1:**
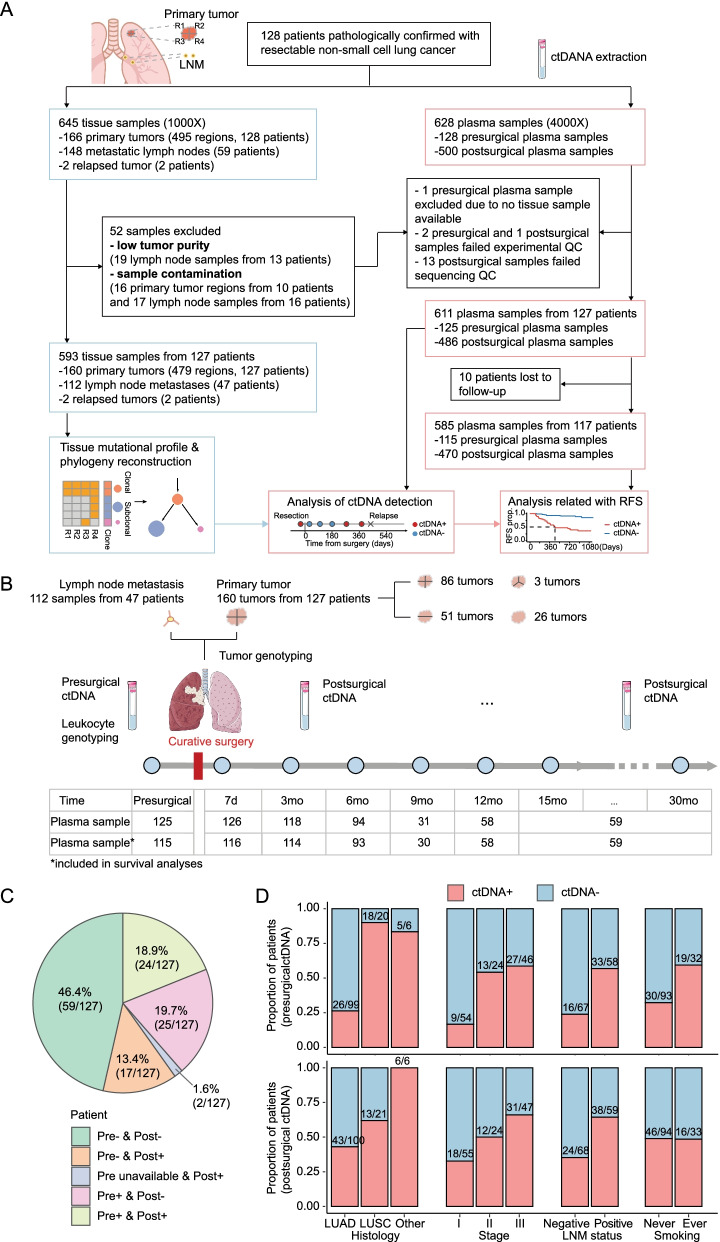
Study design and ctDNA detection. **a** Workflow of sample collection, sample exclusion, and data analysis. **b** Schematic diagram illustrating the timeline for sample collection and the number of plasma samples available for analyses at each time point. **c** Proportions of patients that showed different presurgical and postsurgical ctDNA status. The results of ctDNA detection of all postsurgical samples were included. **d** Proportions of patients positive for presurgical (upper panel) and postsurgical (lower panel) plasma samples, stratified by pathology histology, TNM stage, LNM status, and smoking history

In this study, a total of 128 patients with resectable NSCLC were enrolled (Fig. 1A). Primary tumor and lymph node metastasis (LNM) samples were collected from curative surgeries as standard of care. Plasma samples were collected before surgery, 7 days after surgery, and every three months thereafter. Both tissue and plasma samples were sequenced using a comprehensive 425-gene panel (Fig. 1A, B). One patient was excluded during quality control (Additional files 1, 2: Table S1 and S2).

A total of 611 plasma and 593 tissue samples were included in the analyses (Fig. 1A, Additional file 4: Fig. S1). We reconstructed the clonal phylogeny of each patient from multi-region tissue sequencing to buttress the ctDNA detection (See Additional file 11: Methods). Near half (46.4%, 59/127) of the patients were ctDNA-negative throughout the investigation period. In 32.3% (41/127) of the patients, ctDNA was detected in at least one postsurgical plasma sample, most of whom (65.9%, 27/41) were ctDNA-positive in presurgical samples (Fig. 1C; Additional file 5: Fig. S2).

As shown in Fig. 1D, patients with lung squamous cell carcinoma (LUSC) were more frequently ctDNA-positive than those with lung adenocarcinoma (LUAD). The detection rate correlated with TNM stages and LNM status, and smokers were found with a higher ctDNA-positive rate than non-smokers in presurgical instead of postsurgical results.

**Fig. 2:**
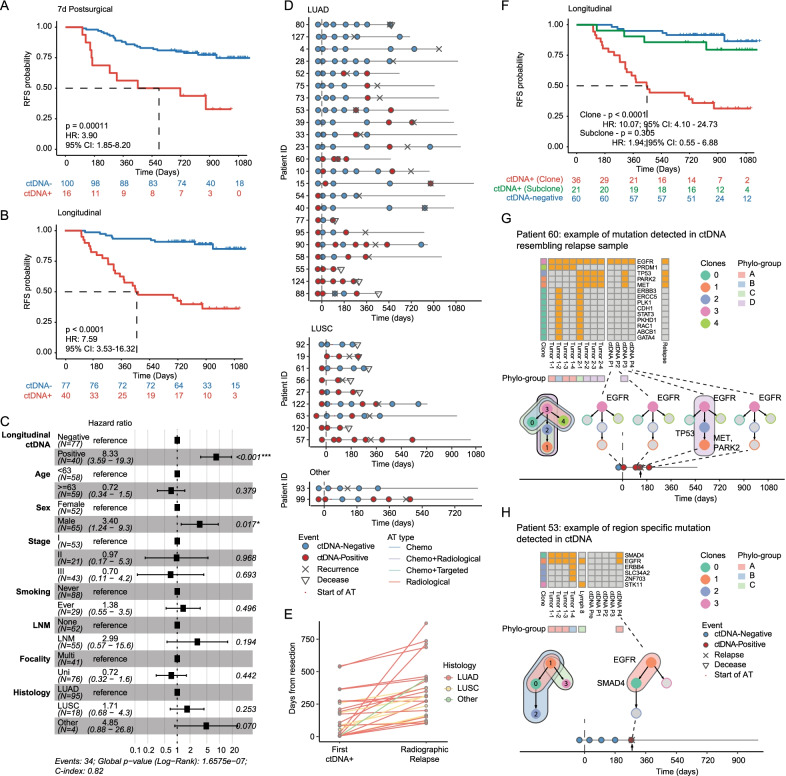
Prognostic values of ctDNA mutation. **a, b** Analysis of recurrence-free survival of patients stratified by 7-day postsurgical (**a**) and longitudinal (**b**) ctDNA detection. Univariate Cox regression results were shown. **c** The results of multivariate-Cox regression for recurrence-free disease in patients stratified by longitudinal ctDNA detection. **d** Swimmer plot illustrating the ctDNA status, adjuvant therapy, and pathological events of cases with disease recurrence (*n* = 34). **e** Time of the earliest ctDNA detection and radiographic relapse, measured by days from the surgery. **f** Analysis of recurrence-free survival of patients stratified by the clonality of longitudinal ctDNA detection. The ctDNA-positive (Clone) group comprised patients with at least one clonal mutation detected in at least one postsurgical plasma sample. The ctDNA-positive (Subclone) group comprised patients with at least one subclonal mutation detected in at least one postsurgical plasma sample and no clonal mutation detected in any postsurgical samples. The ctDNA-negative group comprised patients with no mutation detected in any postsurgical plasma samples. **g, h** Clonal phylogenetic information of tissue and plasma samples of Patient 60 (**g**) and Patient 53 (**h**). Heatmaps denote mutation profiles of multi-regionally resected primary tumors, lymph node metastasis, and plasma samples with clonal annotation (leftmost column) representing mutation clusters. Phylo-groups comprise samples having identical clonal phylogeny. Colored nodes denote the detection of ctDNA mutations in respective clones, whereas gray nodes denote that no mutation in respective clones was detected

Postsurgical ctDNA detection at as early as seven days after surgeries could indicate high risk of recurrence (HR = 3.90, *P* = 0.00011; Fig. 2A), independently of clinicopathological characteristics (multivariate-Cox: HR = 5.49, *P* = 0.002; Fig. 2B). ctDNA detection at following time points (3 months and 6 months) could also serve as prognostic markers (3 months—HR = 4.32, *P* < 0.0001; 6 months—HR = 6.19, *P* < 0.0001) and remained statistically significant after adjusted for clinicopathological characteristics (multivariate-Cox: 3 months—HR = 4.17, *P* < 0.001; 6 month—HR = 4.59, *P* < 0.003; Additional file 6: Fig. S3). Longitudinal ctDNA detection accurately identified high risk of disease recurrence (univariate Cox: HR = 7.59, *P* < 0.0001, Fig. 2B; multivariate-Cox: HR = 8.33, *P* < 0.001, Fig. 2C) and covered the most of relapsed cases (73.5%, 25/34). In these cases, ctDNA detection led radiographic relapse by a median of 145 days. The time intervals were similar between LUAD and LUSC (144 and 150 days, respectively) (Fig. 2D, E; Additional files 7, 8, 9: Figure S4-6). Other results were shown in Additional files 10 and 13.

We further found that clonal mutations in ctDNA were more prognostically informative than subclonal ones. During the longitudinal ctDNA surveillance, patients with clonal mutation exhibited a worse prognosis than ctDNA-negative ones (HR = 10.07, *P* < 0.0001), and no significantly differential survival was observed between those with only subclonal mutations (See Additional file 11: Methods) detected and the ctDNA-negative group (HR = 1.94, *P* = 0.305) (Fig. 2F). Nonetheless, tracking subclonal dynamics in ctDNA may inform the source of relapse. In Patient 60, at the six-month time point, three mutations from subclones 1 and 2 were detected in plasma. Subclones 1 and 2 were specific to three regions of primary tumor 2, suggesting that primary tumor 2 may be the active source of ctDNA. Later the sequencing of the relapse lesion confirmed primary tumor 2 as its clonal origin (Fig. 2G). In Patient 53, clonal *EGFR 19Del* and subclonal *SMAD4* mutations were detected in plasma shortly before disease relapse. The subclonal *SMAD4* mutation was absent from LNM, whereas LNM-specific *STK11* mutation was undetectable in ctDNA, together suggesting that LNM may not be an active source of ctDNA or disease recurrence (Fig. 2H).

In summary, we found that ctDNA could serve as a promising biomarker for risk of recurrence in NSCLC patients who receive curative surgeries, and the results were further discussed in Additional files 3 and 12. As early as 7 days after the surgery, ctDNA detection identified patients at high risk. Longitudinal ctDNA surveillance could reliably predict recurrence, which opens a window of almost 145 days for optimal disease management. Furthermore, our results showed that tracking subclonal dynamics could inform the origin of tumor recurrence.

## Supplementary Information

**Additional file 1. Table S1: **Patient demography.

**Additional file 2. Table S2: **Adjuvant treatment information.

**Additional file 3. Table S3: **Performance of three strategies.

**Additional file 4. Figure S1:** Availability of plasma samples. The availability of plasma samples for analysis at each schedule collection time point. Blue and red blocks denote samples collected before and after disease recurrence, respectively. Abbreviations: LUAD – lung adenocarcinoma, LUSC - lung squamous-cell carcinoma, RFS – recurrence-free survival, AT – adjuvant therapy, RT – radiotherapy, LNM – lymph node metastasis.

**Additional file 5. Figure S2:** Mutational profile of plasma samples. Gene mutations detected in tissue and plasma samples in each patient. Colors denote different variant types. Horizontal and vertical bars denote the detection of tissue mutations in presurgical and postsurgical plasma samples, respectively. Twenty most prevalent gene mutations in tissue samples were shown.

**Additional file 6. Figure S3:** Prognostic values of postsurgical ctDNA detection at 3 months and 6 months. A-B) The recurrence-free survival analysis (top panel) and multi-variant Cox regression (bottom panel) of postsurgical ctDNA detection at 3 months (A) and 6 months (B). For the analysis at 6 months after surgeries, only patients with plasma samples available at this scheduled point and followed-up for more than 6 months were included. Abbreviations: RFS – recurrence-free survival, LNM – lymph node metastasis, LUAD – lung adenocarcinoma, LUSC - lung squamous-cell carcinoma.

**Additional file 7. Figure S4:** ctDNA statuses and disease-related events of patients during follow-up periods. Swimmer plot illustrating the ctDNA statuses, adjuvant therapies, and pathological events of all patients. Abbreviations: LUAD – lung adenocarcinoma, LUSC - lung squamous-cell carcinoma, AT – adjuvant therapy.

**Additional file 8. Figure S5:** Prognostic values of ctDNA detection based on clonal and subclonal mutations. A). The recurrence-free survival analysis of patients stratified by ctDNA detection based on only clonal mutation profiles. B). The recurrence-free survival analysis of patients stratified by ctDNA detection based on all clonal and subclonal mutations.

**Additional file 9. Figure S6:** ctDNA testing, LDCT scans, and disease-related events of patients during follow-up periods. . Swimmer plot illustrating the first positive ctDNA testing, the last negative LDCT scans, and pathological events of patients that experienced recurrence or deceased. B). The original and adjusted time intervals between the first positive ctDNA testing and final LDCT scans that detected disease recurrence. Abbreviations: LDCT – low-dose computed tomography, LUAD – lung adenocarcinoma, LUSC - lung squamous-cell carcinoma.

**Additional file 10. Figure S7:** Prognostic value of presurgical ctDNA detection. A). The recurrence-free survival analysis of patients stratified by presurgical ctDNA detection. B). The multi-variant Cox regression for presurgical ctDNA detection. Abbreviations: RFS – recurrence-free survival, LNM – lymph node metastasis, LUAD – lung adenocarcinoma, LUSC - lung squamous-cell carcinoma.

**Additional file 11. **Methods.

**Additional file 12. **Supplementary discussion.

**Additional file 13. **Supplementary results.

## Data Availability

All relevant data are available in the supplementary materials or on request from the corresponding author for reuse under research purpose only.

## Abbreviations

ctDNA: Circulating tumor DNA
cfDNA: Cell-free DNA
NSCLC: Non-small-cell lung cancer
LUAD: Lung adenocarcinoma
LUSC: Lung squamous cell carcinoma
LNM: Lymph node metastasis
RFS: Recurrence-free survival
HR: Hazard ratio
