# Supplementary figures and images for: Circulating tumor DNA integrating tissue clonality detects minimal residual disease in resectable non-small-cell lung cancer
Source: J Hematol Oncol. 2022 Oct 1;15:137. doi: 10.1186/s13045-022-01355-8 (PMC9526343; doi:10.1186/s13045-022-01355-8)

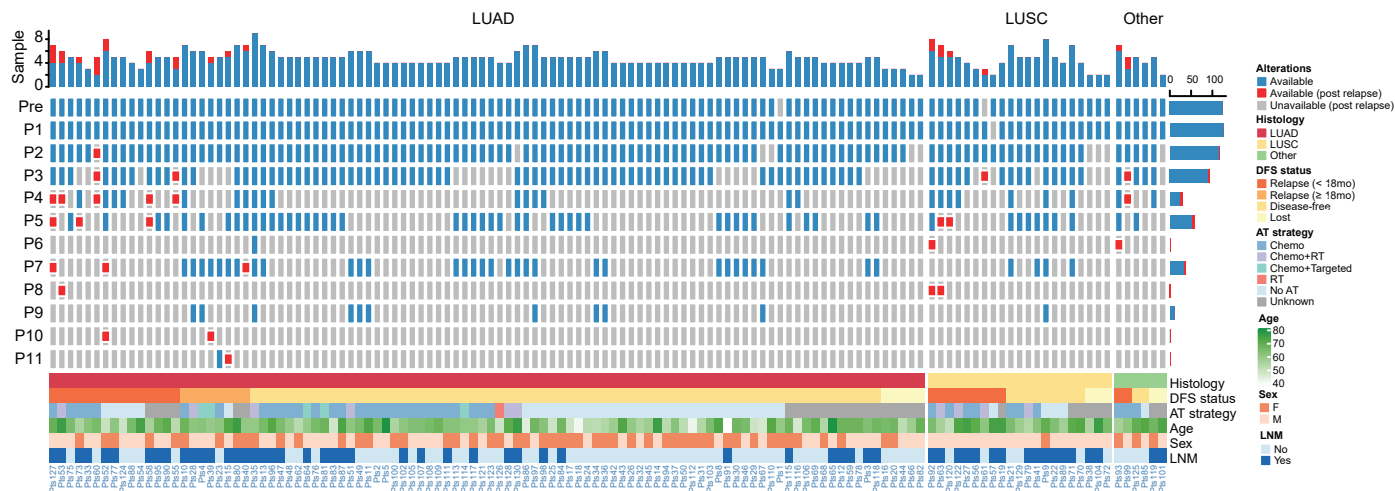

Supplement: Supplementary file 4 — Additional file 4. Figure S1: Availability of plasma samples. The availability of plasma samples for analysis at each schedule collection time point. Blue and red blocks denote samples collected before and after disease recurrence, respectively. Abbreviations: LUAD – lung adenocarcinoma, LUSC - lung squamous-cell carcinoma, RFS – recurrence-free survival, AT – adjuvant therapy, RT – radiotherapy, LNM – lymph node metastasis. [file 13045_2022_1355_MOESM4_ESM.pdf]

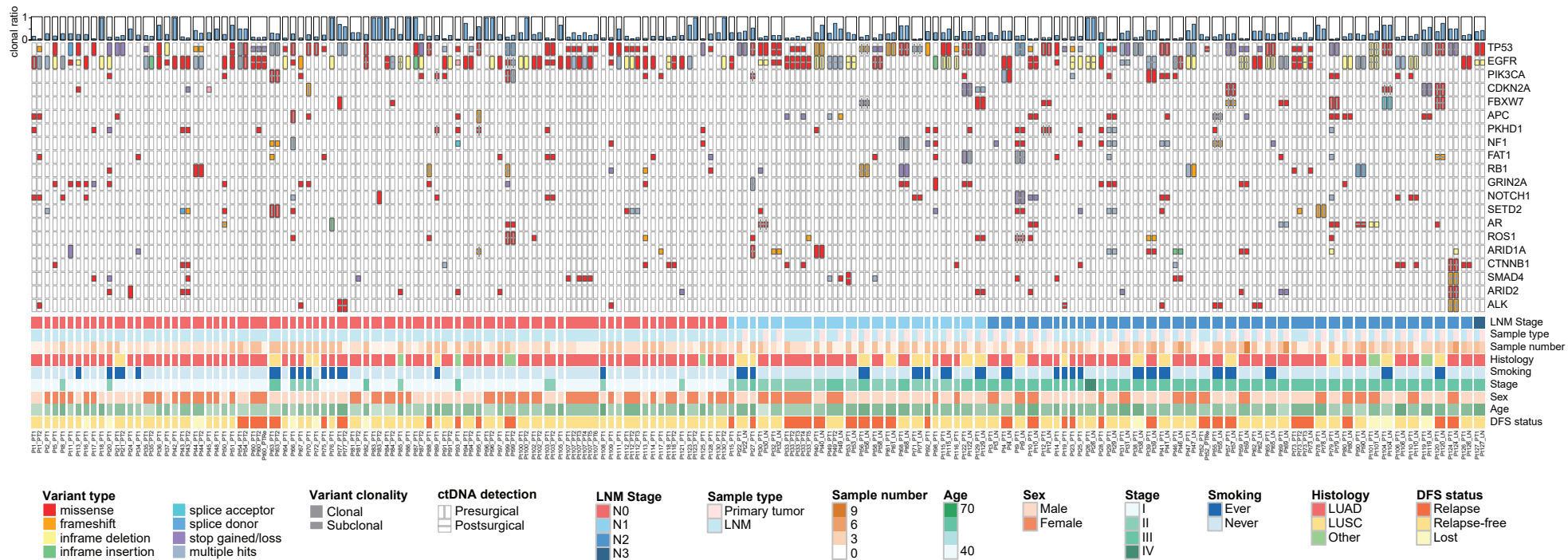

Supplement: Supplementary file 5 — Additional file 5. Figure S2: Mutational profile of plasma samples. Gene mutations detected in tissue and plasma samples in each patient. Colors denote different variant types. Horizontal and vertical bars denote the detection of tissue mutations in presurgical and postsurgical plasma samples, respectively. Twenty most prevalent gene mutations in tissue samples were shown. [file 13045_2022_1355_MOESM5_ESM.pdf]

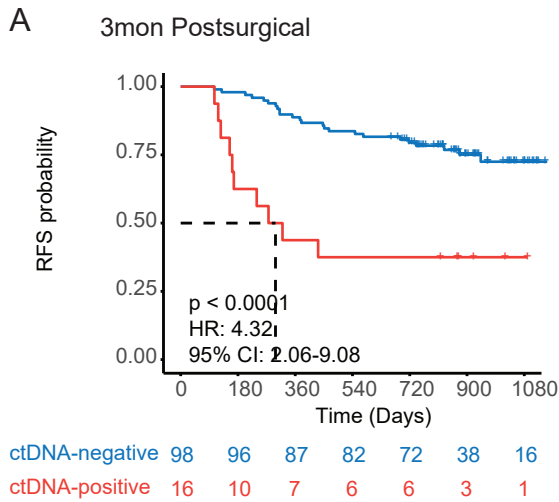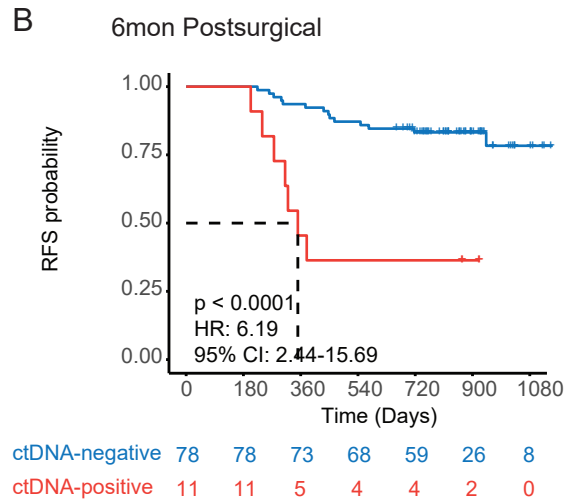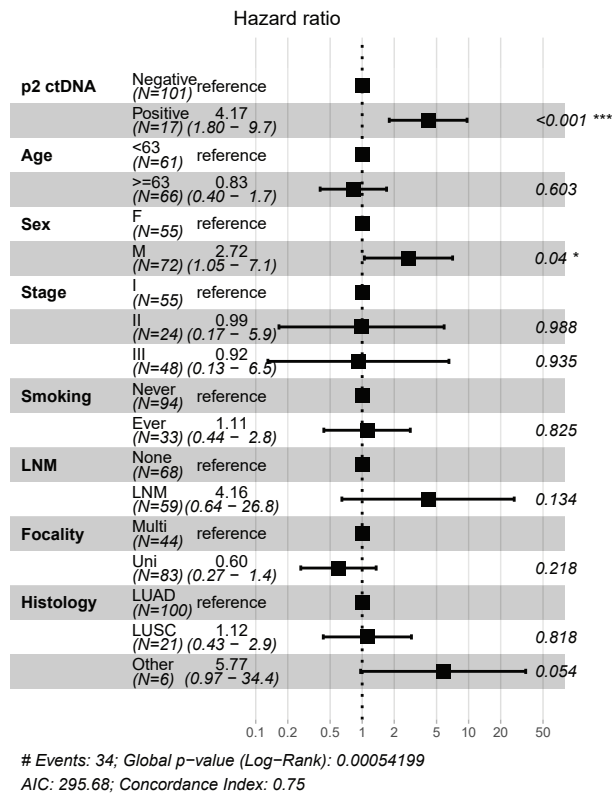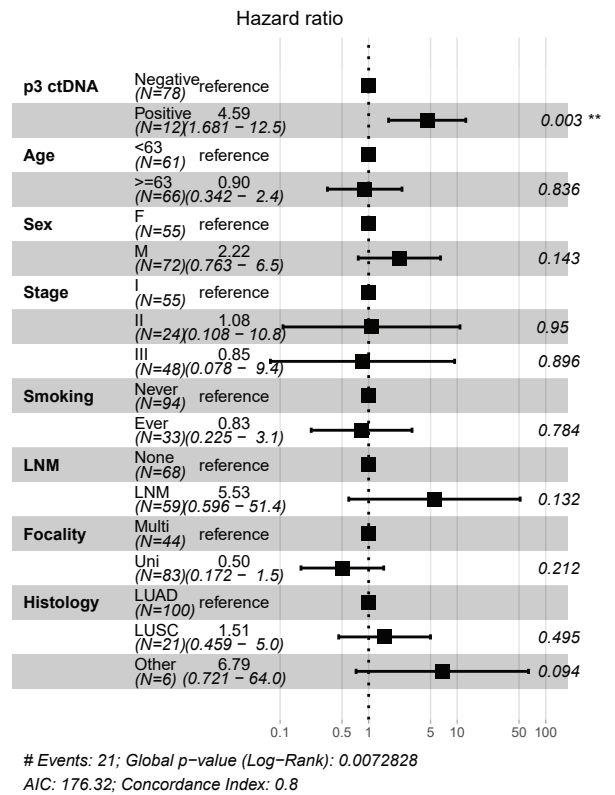

Supplement: Supplementary file 6 — Additional file 6. Figure S3: Prognostic values of postsurgical ctDNA detection at 3 months and 6 months. A-B) The recurrence-free survival analysis (top panel) and multi-variant Cox regression (bottom panel) of postsurgical ctDNA detection at 3 months (A) and 6 months (B). For the analysis at 6 months after surgeries, only patients with plasma samples available at this scheduled point and followed-up for more than 6 months were included. Abbreviations: RFS – recurrence-free survival, LNM – lymph node metastasis, LUAD – lung adenocarcinoma, LUSC - lung squamous-cell carcinoma. [file 13045_2022_1355_MOESM6_ESM.pdf]

## LUAD

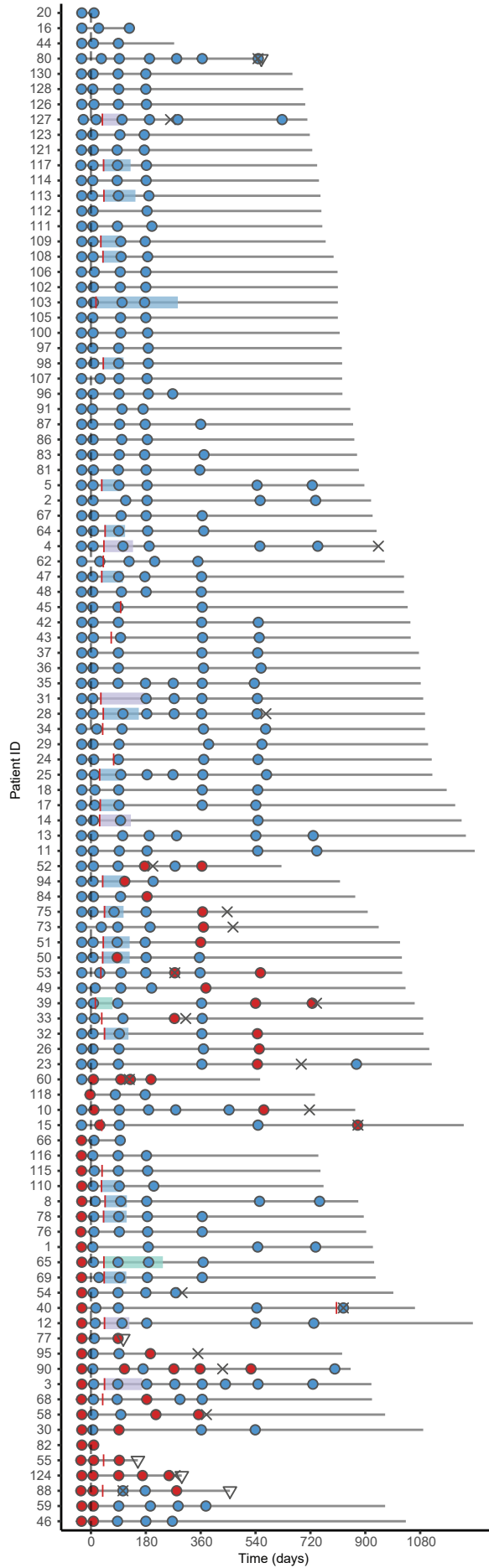

## LUSC

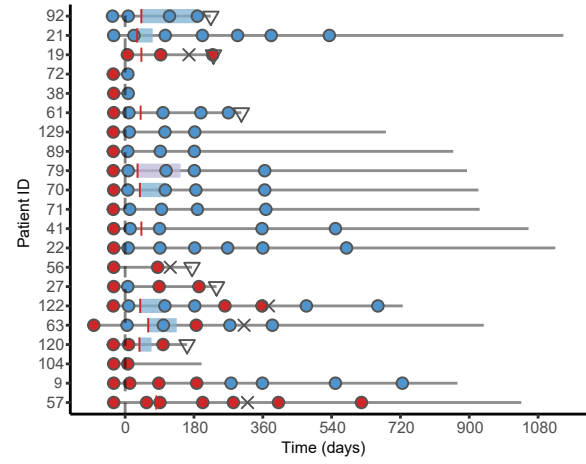

## Other

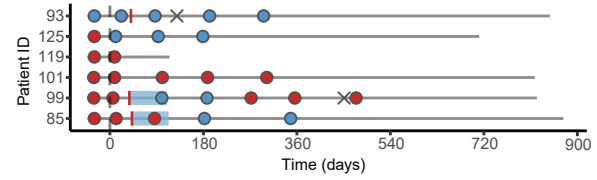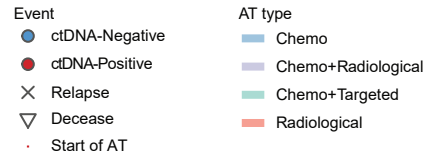

Supplement: Supplementary file 7 — Additional file 7. Figure S4: ctDNA statuses and disease-related events of patients during follow-up periods. Swimmer plot illustrating the ctDNA statuses, adjuvant therapies, and pathological events of all patients. Abbreviations: LUAD – lung adenocarcinoma, LUSC - lung squamous-cell carcinoma, AT – adjuvant therapy. [file 13045_2022_1355_MOESM7_ESM.pdf]

A

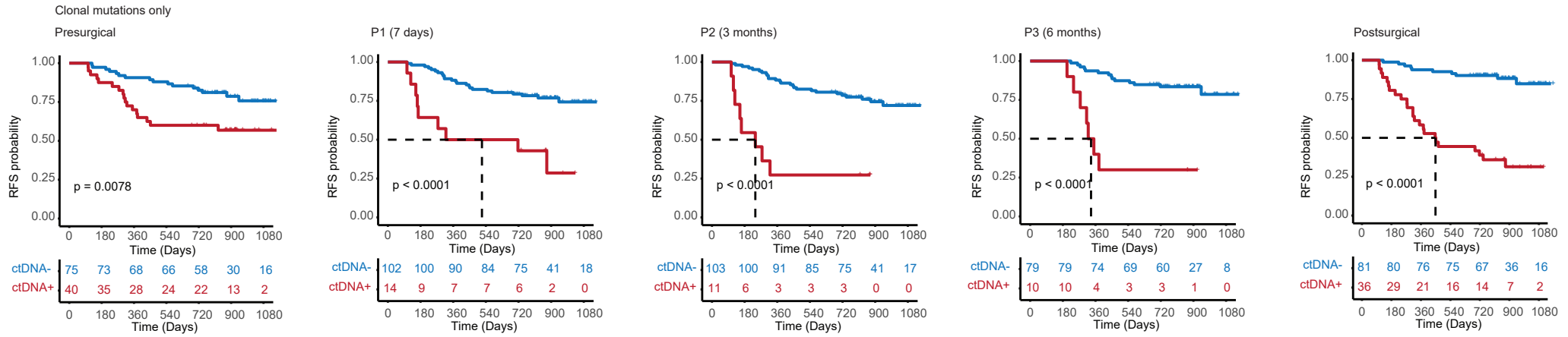

B

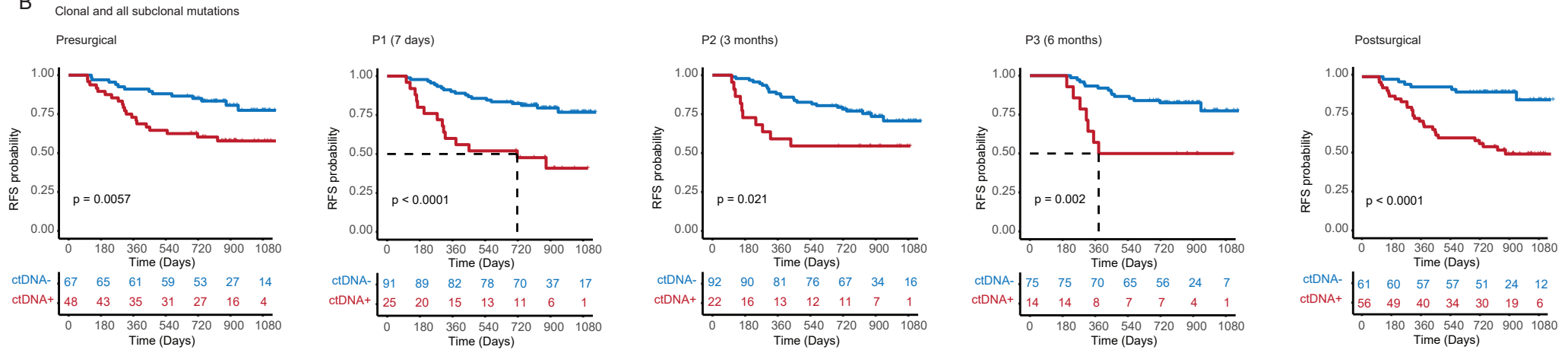

Supplement: Supplementary file 8 — Additional file 8. Figure S5: Prognostic values of ctDNA detection based on clonal and subclonal mutations. A). The recurrence-free survival analysis of patients stratified by ctDNA detection based on only clonal mutation profiles. B). The recurrence-free survival analysis of patients stratified by ctDNA detection based on all clonal and subclonal mutations. [file 13045_2022_1355_MOESM8_ESM.pdf]

A

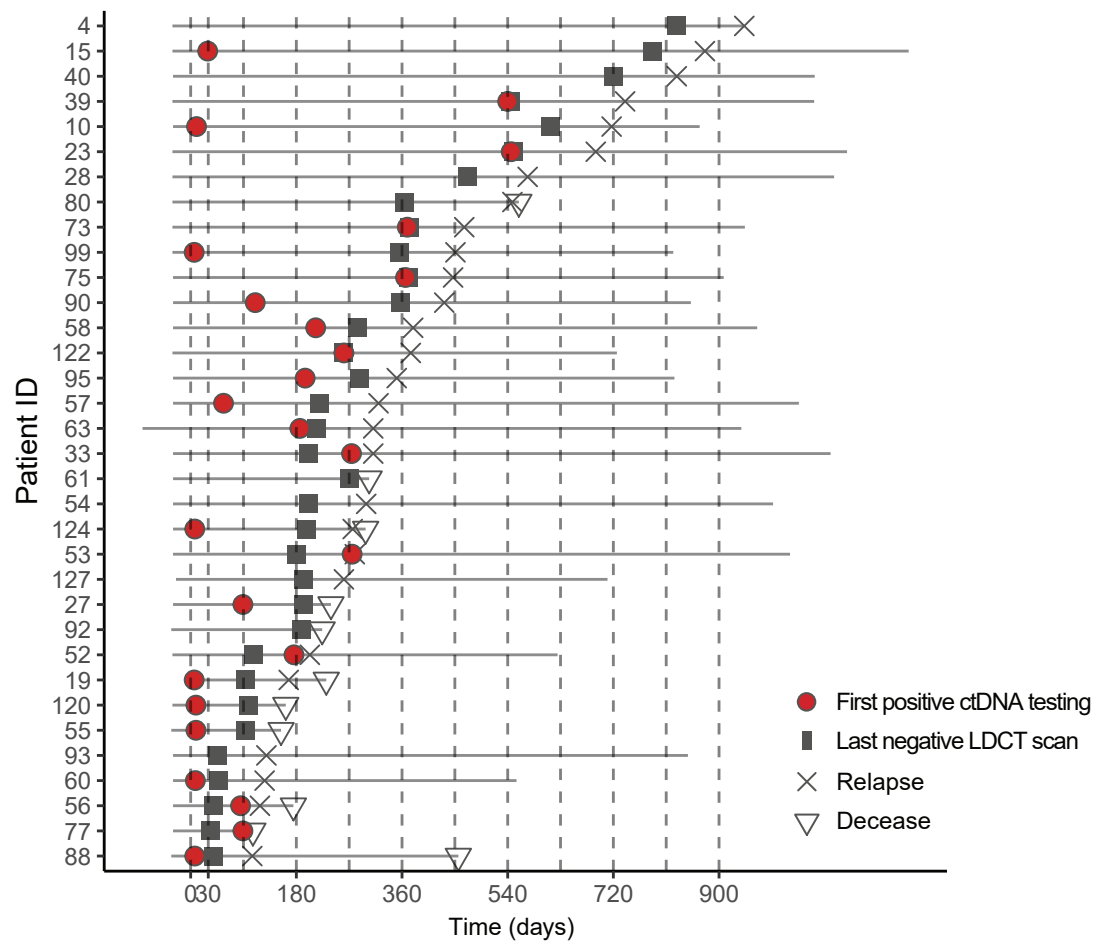

B

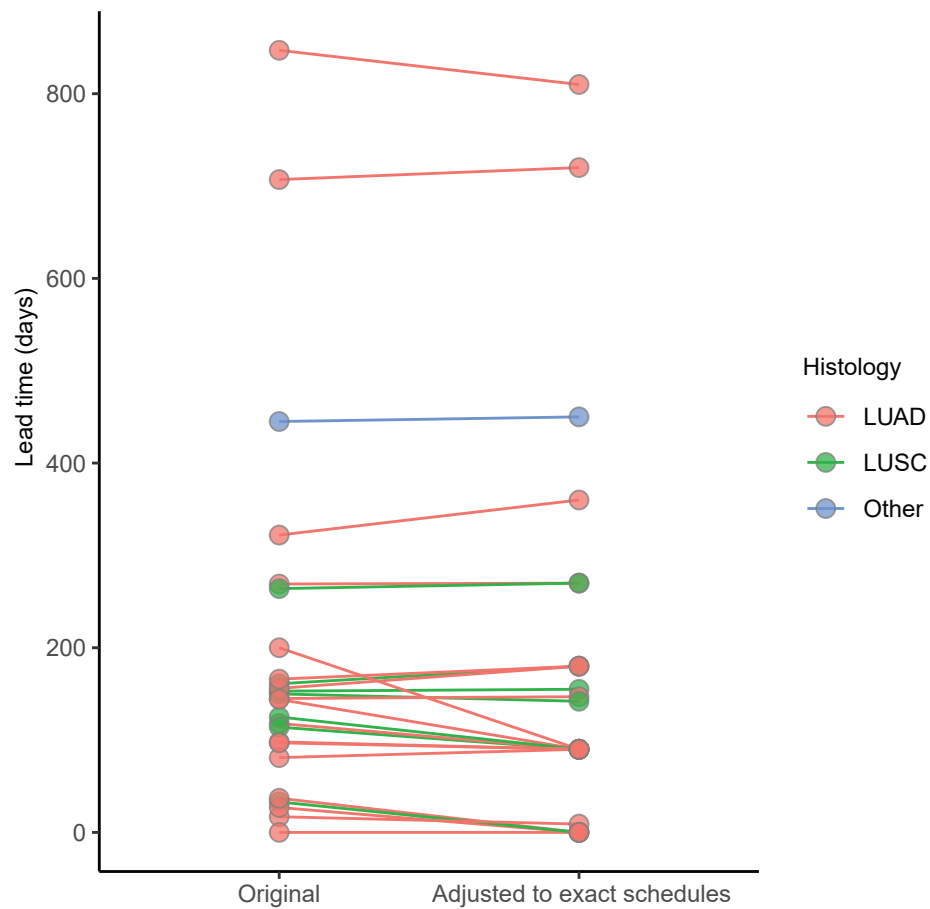

Supplement: Supplementary file 9 — Additional file 9. Figure S6: ctDNA testing, LDCT scans, and disease-related events of patients during follow-up periods. . Swimmer plot illustrating the first positive ctDNA testing, the last negative LDCT scans, and pathological events of patients that experienced recurrence or deceased. B). The original and adjusted time intervals between the first positive ctDNA testing and final LDCT scans that detected disease recurrence. Abbreviations: LDCT – low-dose computed tomography, LUAD – lung adenocarcinoma, LUSC - lung squamous-cell carcinoma. [file 13045_2022_1355_MOESM9_ESM.pdf]

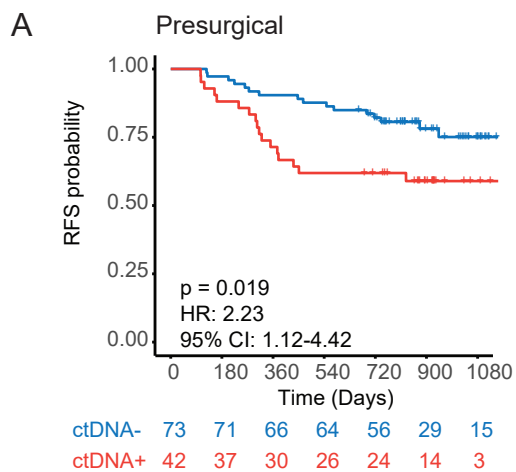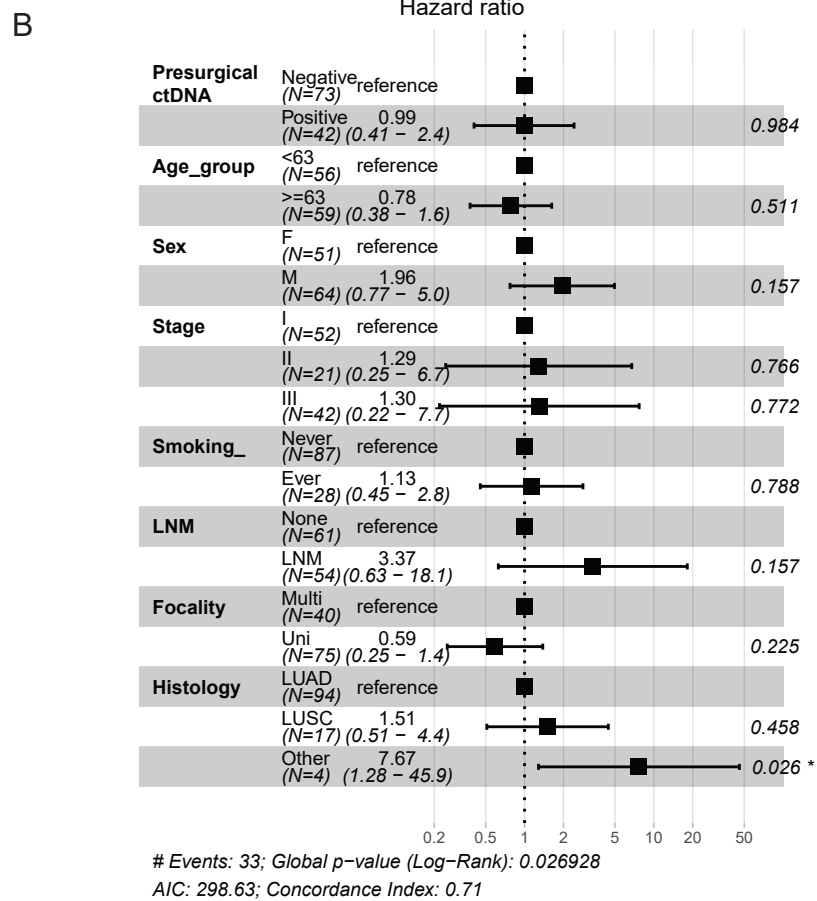

Supplement: Supplementary file 10 — Additional file 10. Figure S7: Prognostic value of presurgical ctDNA detection. A). The recurrence-free survival analysis of patients stratified by presurgical ctDNA detection. B). The multi-variant Cox regression for presurgical ctDNA detection. Abbreviations: RFS – recurrence-free survival, LNM – lymph node metastasis, LUAD – lung adenocarcinoma, LUSC - lung squamous-cell carcinoma. [file 13045_2022_1355_MOESM10_ESM.pdf]
