# Supplementary material for: Circulating tumor DNA integrating tissue clonality detects minimal residual disease in resectable non-small-cell lung cancer
Source: J Hematol Oncol. 2022 Oct 1;15:137. doi: 10.1186/s13045-022-01355-8 (PMC9526343; doi:10.1186/s13045-022-01355-8)
Supplement: Supplementary file 11 — Additional file 11. Methods. [file 13045_2022_1355_MOESM11_ESM.docx]

**Title:** Circulating tumor DNA integrating tissue clonality detects minimal residual disease in resectable non-small-cell lung cancer

**Authors:** *Wang* et al.

**Methods**

**Patient and sample collection**

Patients enrolled in this study belong to a prospective cohort study (a non-interventional systematic study for the Non-Small-Cell Lung Cancer Tempo-spatial Heterogeneity (LuCaTH); ChiCTR1900022521, http://www.chictr.org.cn/showproj.aspx?proj=34204). All 128 patients enrolled had not received neoadjuvant therapy and were pathologically confirmed with NSCLC (AJCC, 8th edition). Curative tumor resections were performed as the standard of care. All patient samples were identified and reviewed by two pathologists to determine the histological subtype and TNM staging (**Table S1, S2**). One patient was later excluded from ctDNA surveillance due to no tissue sample available after quality control processes. For the rest 127 patients, a total of 645 tumor tissue samples were initially collected and sequenced with a panel covering 425 cancer-associated genes. Fifty-two samples were excluded due to low tumor purity or DNA contamination, resulting in 593 tissue samples available for analysis. Presurgical plasma and leukocyte control samples were collected 1-3 days before surgeries with one exception of 52 days. Postsurgical plasma sample collections were scheduled at 7 days after surgeries and every 3 months thereafter. Each patient was serially monitored for at least two time points after surgeries unless the patient deceased or was lost to follow-up before the schedule date of collection. A total of 627 plasma samples and 127 leukocyte samples were initially collected. Two presurgical plasma samples and one 7-day postsurgical plasma sample were excluded due to failure in quality control processes before sequencing. The remaining 624 plasma samples and 127 leukocyte samples were sequenced with the same gene panel as tissue samples. Thirteen plasma samples were excluded due to DNA contamination, resulting in a final number of 611 plasma samples and 127 leukocyte samples available for analysis (**Figure 1A, 1B, S1**). The institutional review board of Jiangsu Cancer Hospital approved the study, and all the patients provided written informed consent.

**Library preparation and sequencing**

For targeted sequencing, genomic DNA from tissue samples was extracted with QIAamp DNA FFPE Tissue Kit; genomic DNA from plasma and leukocyte samples was extracted with DNeasy Blood & Tissue Kit (Qiagen). Extracted DNA was quantified by Qubit 3.0 using the dsDNA HS Assay Kit (ThermoFisher Scientific), following the manufacturer’s instruction. FFPE samples were de-paraffinized with xylene followed by genomic DNA extraction using QIAamp DNA FFPE Tissue Kit (Qiagen) following the manufacturer’s instruction. Plasma samples were first centrifuged at high speed to remove any cell debris, followed by cell-free DNA (cfDNA) extraction from the supernatant using QIAamp Circulating Nucleic Acid Kit (Qiagen).

Library preparations were performed with KAPA Hyper Prep Kit (KAPA Biosystems) according to manufacturer’s suggestions for different sample types. In brief, sequential operations of end-repairing, A-tailing, and indexed adapter ligation were performed to 6.08 - 200 ng (median: 70.5 ng) of cfDNA or 1 μg of fragmented genomic DNA, followed by size selection using Agencourt AMPure XP beads (Beckman Coulter). Hybridization-based target enrichment was carried out with GeneseeqPrimeTM pan-cancer gene panel (425 cancer-relevant genes), and xGen Lockdown Hybridization and Wash Reagents Kit (Integrated DNA Technologies). Captured libraries were on-beads PCR amplified with Illumina p5 (50 AAT GAT ACG GCG ACC ACC GA 30) and p7 primers (50 CAA GCA GAA GAC GGC ATA CGA GAT 30) in KAPA HiFi HotStart ReadyMix (KAPA Biosystems), followed by quantification by qPCR using the KAPA Library Quantification Kit (KAPA Biosystems) and purification using Agencourt AMPure XP beads. Library fragment size was assessed by Bioanalyzer 2100 (Agilent Technologies). Sequencing of the target-enriched library was then performed on Illumina HiSeq4000 platform using PE150 sequencing chemistry (Illumina). The average coverage depth was 902X, 4421X, 261X for tissue, plasma, and leukocyte samples, respectively.

**Single nucleotide variant and insertion/deletion calling in tissue samples**

Trimmomatic was used for FASTQ file quality control. Leading/trailing low quality (quality reading below 20) or N bases were removed. Paired-end reads were then aligned to the reference human genome (build hg19), using the Burrows–Wheeler Aligner (BWA, v0.7.12) [1]. Local realignment around indels and base quality score recalibration was performed with the Genome Analysis Toolkit (GAT1K 3.4.0) [2]. Matched tumor and normal leukocyte sample pairs were first checked to have the same SNP fingerprint using VCF2LR (GeneTalk, https://web.gene-talk.de/) and nonmatching samples were removed from analysis. Further, samples with mean dedup depth <100X were removed. Cross-sample contamination was estimated using ContEst (Broad Institute) [3]. Briefly, ContEst quantifies contamination in next-generation sequencing data by identifying homozygous non-reference SNPs in the 1000 Genomes database and assessing the likelihood of observing alternate alleles at these genomic locations in the sequencing data. Somatic Single Nucleotide Variant (sSNV) and insertion/deletion (InDel) calling was performed using Vardict [4]. For tissue samples, SNVs and InDels called were further filtered using the following criteria: (i) filtered if variant supporting reads <5 or variant allele frequency (VAF) supporting the variant < 2%, (ii) filtered if present in >1% population frequency in the 1000 Genomes or ExAC database, (iii) filtered through an internally collected list of recurrent sequencing errors (≥3 variant reads and ≤20% VAF in at least 30 out of ~2,000 normal samples) on the same sequencing platform.

**Reconstruction of clonal phylogenetics of tissue samples and clonality-based filtering**

ABSOLUTE [5] was used to infer tumor purity, taking segmented copy number data from FACETS [6] with allelic fractions of somatic point mutations from the tissue mutation calling step. The copy number estimates from FACETS, tumor purity from ABSOLUTE, and allelic counts of somatic mutations from the Mutation Calling step were then fed to PyClone (v.0.13.0) [7] to infer tumor clones and their fractions. PyClone was run with 1000 iterations and a burn-in of 1000. Default settings were used for other parameters: alpha = 1, beta = 1, concentration = 1, prior shape = 1.0, and rate = 0.001. Clusters with cancer cell fractions (CCFs) greater than 0.02 in a sample were considered truly present and used for phylogeny reconstruction.

Phylogenetic trees of clones (subclonal hierarchies) were reconstructed by SCHISM with estimated somatic mutation CCFs across tumor samples from PyClone as inputs. SCHISM (v.1.1.2) [8] was run in the Sequential Mode, with the K-means algorithm. Default settings were used for other parameters: generation count = 50, generation size = 1000, random object fraction = 0.2, mutation probability = 0.9, and fitness coefficient = 5.0. Mutations falling in the truncal clone were classified as clonal. If the truncal clone consisted of only one single mutation and the CCF of its direct descendent clone exceeded 80% of its CCF, mutations falling in this descendent clone was also considered clonal. All the other mutations were classified as subclonal.

**Variant calling and polishing in plasma samples**

Variant calling of plasma samples was tumor-informed. The tissue mutational profile of an individual was composed of any mutations detected in the primary tumor and paired LNM samples, if available. Tissue clonality was annotated to each variant detected in plasma. SNVs and InDels detected in plasma samples were filtered if (i) they were not in the paired tissue mutational profile; (ii) they fell in an in-house list of clonal hematopoiesis (CH) variants; or (iii) they were detected in paired leukocyte controls with at least one variant read. In addition, we collected plasma samples from 31 healthy individuals to establish a normal control pool to further eliminate stereotypical errors in alignment data from independent plasma samples. The method was modified from the one initially created and described by Newman, Aaron M., et al. [9]. For each variant (SNV or InDel) detected in patient tissue and plasma samples, its abundance in the ith (i = 1, …, 31) normal sample was calculated as fi = N_alt_(i) / (N_ref_(i) + N_alt_(i)), where N_alt_(i) and N_ref_(i) were the numbers of alteration-supporting reads and reference reads, respectively. F = [fi] (i = 1, …, 31) was defined as the background distribution of this variant. The abundance of a candidate variant in a plasma sample was calculated as AF = N*_alt_ / (N*_ref_ + N*_alt_), where N*_alt_ and N*_ref_ were the numbers of alteration-supporting reads and reference reads, respectively, in this plasma sample. If the corresponding background distribution F was Gaussian, we evaluated AF with a one-sided z-test, yielding a p-value. Otherwise, a Zero-Inflated Weibull-Poisson (ZIWP) distribution was fit using the F values, yielding parameters, the shape, scale, and fraction δ of non-zero values. We used the estimated shape and scale parameters to calculate the cumulative probability P_0_ that a given value sampled from the ZIWP model was below AF. We then adjust the cumulative probability P_0_ using δ to obtain the P value, P = δ (1 - P_0_). The resulting P values were then adjusted for multiple hypotheses using a stringent Bonferroni correction (where n is the number of variants tested with the normal control pool). We examined all candidate variants that occurred in more than 1 normal control and eliminated the given candidate if its abundance AF was statistically indistinguishable from the background (FDR-adjusted P ≥ 0.01). To maximize the prognostic value of ctDNA detection, all plasma samples with clonal mutations or subclonal mutations of VAF greater than 0.5% detected were defined as ctDNA-positive, otherwise ctDNA-negative.

**Assay validation for limit of blank**

Two reference cell lines (NA19240 and NA18535) from National Institute of Standards and Technology (NIST) were used to assess the analytical specificity to confirm that our quality metrics and filtering criteria minimized the false positive rate. We mixed cDNA from two reference cell lines and created a gradient series of DNA input at 200ng, 100ng, 50ng, 25ng, and 10ng. Specificity was observed at 100% with no unverified mutations reported across 3 replicates for each amount of DNA input.

**Statistical analysis**

Comparisons between continuous numeric data were done using the Wilcoxon test. Comparisons of proportion between groups were done using the Fisher's exact test. For survival analyses, Kaplan-Meier curves were compared using the log-rank test; hazard ratios (HRs) were calculated by Cox proportional hazards model. A two-sided *P* value of less than 0.05 was considered significant for all tests unless indicated otherwise. All statistical analyses were done in R (v4.0.2).

**References**

1. Li H, Durbin R: **Fast and accurate short read alignment with Burrows-Wheeler transform.** *Bioinformatics* 2009, **25:**1754-1760.

2. McKenna A, Hanna M, Banks E, Sivachenko A, Cibulskis K, Kernytsky A, Garimella K, Altshuler D, Gabriel S, Daly M, DePristo MA: **The Genome Analysis Toolkit: a MapReduce framework for analyzing next-generation DNA sequencing data.** *Genome Res* 2010, **20:**1297-1303.

3. Cibulskis K, McKenna A, Fennell T, Banks E, DePristo M, Getz G: **ContEst: estimating cross-contamination of human samples in next-generation sequencing data.** *Bioinformatics* 2011, **27:**2601-2602.

4. Lai Z, Markovets A, Ahdesmaki M, Chapman B, Hofmann O, McEwen R, Johnson J, Dougherty B, Barrett JC, Dry JR: **VarDict: a novel and versatile variant caller for next-generation sequencing in cancer research.** *Nucleic Acids Res* 2016, **44:**e108.

5. Carter SL, Cibulskis K, Helman E, McKenna A, Shen H, Zack T, Laird PW, Onofrio RC, Winckler W, Weir BA, et al: **Absolute quantification of somatic DNA alterations in human cancer.** *Nat Biotechnol* 2012, **30:**413-421.

6. Shen R, Seshan VE: **FACETS: allele-specific copy number and clonal heterogeneity analysis tool for high-throughput DNA sequencing.** *Nucleic Acids Res* 2016, **44:**e131.

7. Roth A, Khattra J, Yap D, Wan A, Laks E, Biele J, Ha G, Aparicio S, Bouchard-Cote A, Shah SP: **PyClone: statistical inference of clonal population structure in cancer.** *Nat Methods* 2014, **11:**396-398.

8. Niknafs N, Beleva-Guthrie V, Naiman DQ, Karchin R: **SubClonal Hierarchy Inference from Somatic Mutations: Automatic Reconstruction of Cancer Evolutionary Trees from Multi-region Next Generation Sequencing.** *PLoS Comput Biol* 2015, **11:**e1004416.

9. Newman AM, Lovejoy AF, Klass DM, Kurtz DM, Chabon JJ, Scherer F, Stehr H, Liu CL, Bratman SV, Say C, et al: **Integrated digital error suppression for improved detection of circulating tumor DNA.** *Nat Biotechnol* 2016, **34:**547-555.
