# Supplementary material for: Circulating tumor DNA integrating tissue clonality detects minimal residual disease in resectable non-small-cell lung cancer
Source: J Hematol Oncol. 2022 Oct 1;15:137. doi: 10.1186/s13045-022-01355-8 (PMC9526343; doi:10.1186/s13045-022-01355-8)
Supplement: Supplementary file 12 — Additional file 12. Supplementary discussion. [file 13045_2022_1355_MOESM12_ESM.docx]

**Title:** Circulating tumor DNA integrating tissue clonality detects minimal residual disease in resectable non-small-cell lung cancer

**Authors:** *Wang* et al.

**Supplementary Discussion**

Our study was different from other published studies in multiple aspects – 1). Our study included 645 tissue samples from 127 patients. Each primary tumor was sectioned into multiple regions. The multi-region profiling identified more subclonal mutations and enabled accurate and reliable reconstruction of the tumor clonality. 2). Our assay employed the tissue clonality information to increase the sensitivity while controlling for the false positive rate in the ctDNA detection. Different from studies that considered only clonal mutations, we also evaluated subclonal mutations of high confidence, which helped the assay achieve similar levels of performance (sensitivity: 73.5%, specificity: 83.1%) in monitoring disease recurrence. 3). We scheduled the first postsurgical cfDNA testing at as early as 7 days after the surgery, when patients were ready for hospital discharge. Our results showed that the 7th-day testing was able to identify a group of patients (10/33, 30.3%) at high risk of recurrence. The timeframe may improve patient adherence and provide a time window for the early planning of disease management. 4). The sequencing depth we used for plasma samples was lower (~4,000X) than those used in other studies, such as ~40,000X in Abbosh et al.’s study[1], ~30,000X in Qiu et al.’s study[2], and ~10,000X in Chaudhuri et al.’s study[3]. The cost for plasma sequencing was lower in our study. 5). We used a 425-gene panel that had been widely used in the tissue sequencing of multiple cancer types. Other studies either used bespoke liquid biopsy panels for lung cancer or customized panels for each individual. Our larger panel allowed for the detection of rare mutations that were less prevalent in lung cancer populations. Using the same panel also provided convenience and cost-effectiveness when incorporating this liquid biopsy assay into the clinical genomic testing system in use.

Intratumor heterogeneity has significant influence on the diagnosis, planning of treatments, prediction of prognosis, and mechanism understanding of metastasis and recurrence in lung cancer. The overall proportion of subclonal mutations may not be associated with postsurgical relapse. However, certain subclonal mutations could made up “branches” and drive tumor progression and/or recurrence. Therefore, it is of clinical significance to accurately characterize tumor clonality. Multi-region sequencing is one of the most successful strategies in investigating intratumor heterogeneity and inferring clonal evolution[4]. We used a 425-gene panel to profile at least two regions of each tumor, making it possible to detect both driver and passenger mutations, including a number of region-specific ones. The breadth of the mutational profile of tissue samples allowed for stable and reliable inference of the clonal architecture in each patient.

Our method tracked both clonal mutations and subclonal mutations for monitoring the MRD status. Stringent filtering processes were applied to all mutations to control for the technical false positive rate. Subclonal mutations went through an additional filtering by the allelic frequency (AF > 0.5%) in plasma to identify prognostically meaningful ones. On the one hand, we validated the findings illustrated in some other studies[1, 2] that tracking clonal mutations in plasma could help identify MRD statuses after definitive treatments (**Fig. S5**). On the other hand, we showed that most subclonal mutations may not be prognosis-associated (**Fig. 2F**) whereas high-AF ones could identify individuals at high risk of recurrence independently of clonal mutations (**Table S3**).

Specifically, high-AF subclonal mutations identified three recurrence cases (P19, P90, and P99) at 7th-day and 3rd-month time points in the absence of clonal mutations (**Fig. S4; Table S3**). In patient P90, only subclonal *AR (p.Q62L)* mutation was detectable throughout the postsurgical period. The disease recurred 426 days after the first detection of the AR mutation. In patient P19, subclonal *ZNF703 (p.A513-A514del)* mutation was first detected six days after the surgery and remained positive after three months. The patient experienced rapid relapse at 167 days, only after which were clonal mutations detectable in the plasma. Patient P99 had subclonal *GNAS (p.S455-D466del)* mutation detected at the 6th day, which disappeared following the administration of chemotherapy. The patient remained disease-free until 445 days later. Results of these cases implied for the significance of tracking high-AF subclonal mutations.

Patient P60 was the only case where relapse tissue sample sequencing was available. The patient had an additional ctDNA testing during the visit when the recurrence was detected by low-dose computed tomography (LDCT), which detected subclonal mutations that associated the plasma sample with the relapse sample and a region of the primary lesion. This case suggested that tracking high-AF subclonal mutations may also provide the information of the origin of disease recurrence.

**References**

1. Abbosh C, Birkbak NJ, Wilson GA, Jamal-Hanjani M, Constantin T, Salari R, Le Quesne J, Moore DA, Veeriah S, Rosenthal R, et al: **Phylogenetic ctDNA analysis depicts early-stage lung cancer evolution.** *Nature* 2017, **545:**446-451.

2. Qiu B, Guo W, Zhang F, Lv F, Ji Y, Peng Y, Chen X, Bao H, Xu Y, Shao Y, et al: **Dynamic recurrence risk and adjuvant chemotherapy benefit prediction by ctDNA in resected NSCLC.** *Nat Commun* 2021, **12:**6770.

3. Chaudhuri AA, Chabon JJ, Lovejoy AF, Newman AM, Stehr H, Azad TD, Khodadoust MS, Esfahani MS, Liu CL, Zhou L, et al: **Early Detection of Molecular Residual Disease in Localized Lung Cancer by Circulating Tumor DNA Profiling.** *Cancer Discov* 2017, **7:**1394-1403.

4. Senosain MF, Massion PP: **Intratumor Heterogeneity in Early Lung Adenocarcinoma.** *Front Oncol* 2020, **10:**349.
