# Supplementary material for: Circulating tumor DNA integrating tissue clonality detects minimal residual disease in resectable non-small-cell lung cancer
Source: J Hematol Oncol. 2022 Oct 1;15:137. doi: 10.1186/s13045-022-01355-8 (PMC9526343; doi:10.1186/s13045-022-01355-8)
Supplement: Supplementary file 13 — Additional file 13. Supplementary results. [file 13045_2022_1355_MOESM13_ESM.docx]

**Title:** Circulating tumor DNA integrating tissue clonality detects minimal residual disease in resectable non-small-cell lung cancer

**Authors:** *Wang* et al.

**Supplementary results**

**Patient characteristics**

The cohort had a median age of 63 years (range: 39 – 79 years). There were 72 males (56.7%) and 55 females (43.3%). The majority (78.7%, 100/127) of patients had adenocarcinomas compared to 16.5% (21/127) having squamous cell carcinomas, and 4.8% (6/127) having other types of patho-histology. The majority (43.3%, 55/127) of patients had stage I diseased, with 18.9% (24/127) having stage II, 37.0% (47/127) at stage III, and one person later restaged to IV. The majority (74.0%, 94/127) of patients were never smokers, and the rest (26.0%, 33/127) had histories of smoking. Ten patients were lost to follow-up, all of whom had at least one postsurgical plasma sample though, resulting in 117 patients available for survival analysis. Thirty-four (29.06%) patients experienced recurrence of disease at a median of 311 days after surgeries (range: 105 – 943 days). The median follow-up period for the recurrence-free patients was 900 days after surgeries (range: 661 – 1259 days) (**Table S1**).

**Prognostic value of presurgical ctDNA detection**

Pre-surgical ctDNA detection showed the potential to identify patients at high risk when analyzed solely (HR = 2.23, 95% CI = 1.12 - 4.42, *P* = 0.019) (**Fig. S7A**). However, it failed to retain statistical significance after adjusted for clinicopathological characteristics in the multivariate analysis (P = 0.984) (**Fig. S7B**).
